# Predictive value of CD3^+^ cells and interleukin 2 receptor in systemic inflammatory response syndrome after percutaneous nephrolithotomy

**DOI:** 10.3389/fimmu.2022.1017219

**Published:** 2022-11-24

**Authors:** Yu He, Ding Xia, Yonghua Tong, Haojie Shang, Xiao Liu, Ejun Peng, Qiu Huang, Kun Tang, Zhiqiang Chen

**Affiliations:** Department of Urology, Tongji Hospital, Tongji Medical College, Huazhong University of Science and Technology, Wuhan, China

**Keywords:** CD3^+^ cells, IL-2R, nomogram, percutaneous nephrolithotomy, systemic inflammatory response syndrome

## Abstract

**Objective:**

The aim of the current study was to evaluate the risk factors that influence the development of postoperative systemic inflammatory response syndrome (SIRS) after percutaneous nephrolithotomy (PCNL), including cytokines and lymphocyte subsets.

**Methods:**

A total of 154 patients who underwent PCNL at our hospital between October 2019 and January 2022 were retrospectively reviewed. The development of post-PCNL SIRS was the primary endpoint of the study. Univariable analysis and multivariable logistic regression analysis were performed to identify independent risk factors of post-PCNL SIRS. A nomogram was constructed using the independent risk factors, and receiver operating characteristic (ROC) curves were drawn.

**Results:**

There were 50 patients (32.5%) who developed SIRS after PCNL. In multivariate analysis, positive urine culture (odds ratio [OR], 3.556; *p* = 0.048), long operation time (OR, 1.011; *p* = 0.027), high IL-2R (OR, 1.002; *p* = 0.018), low percentage of CD3^+^ cells (OR 0.931; *p* = 0.006), and high white blood cell (WBC) count (OR, 1.282*; p* = 0.044) were independent risk factors for post‐PCNL SIRS. These five significant variables were used to generate a nomogram that exhibited favorable fitting. The discrimination area under the ROC curves was 0.795.

**Conclusions:**

Patients with long operation times, positive urine cultures, high interleukin 2 receptor, high white blood cell counts, and low percentages of CD3^+^ cells may be at a higher risk of developing SIRS after PCNL. In these patients, cautious and comprehensive preoperative evaluations and appropriate treatment strategies should be considered.

## Introduction

The incidence of nephrolithiasis ranges from 1% to 13% globally (1). More than half of the patients relapse within 10 years (2). There are a variety of treatment methods for nephrolithiasis of different sizes and locations. Due to the advantages of PCNL such as high stone‐free rates and quick recovery, PCNL has become the first-line treatment for patients with staghorn calculi, complex stones, and most upper urinary tract stones >20 mm in diameter. Notably, however, there are many postoperative complications associated with PCNL. Sepsis is one of the most common and serious complications and is defined as a life-threatening organ dysfunction caused by a dysregulated host response to infection (3, 4). SIRS is considered the first step in the cascade reaction of sepsis and is strongly associated with the development of sepsis (5). The rate of post-PCNL SIRS ranges from 22.1% to 43.0% in previous studies (6, 7).

Previous studies indicated that C-reactive protein, operation time, stone culture, and stone burden are risk factors for post-PCNL SIRS (8, 9). However, their predictive value has been inconsistent across studies. Some of these indicators have drawbacks, such as stone culture and stone composition analysis, which are difficult to assess preoperatively. Thus, it is vital to identify additional efficient markers that can accurately predict the occurrence of post-PCNL SIRS.

Many studies have demonstrated that cytokine and lymphocyte subsets are correlated with inflammation (10–12). During immune responses, cytokines and lymphocyte subsets play important roles by regulating immune responses (13, 14). Interleukin (IL)-6 and IL-1β were identified to be associated with neuroinflammatory diseases (15). IL-8 and tumor necrosis factor (TNF) α are involved in important pathophysiological processes in inflammatory bowel disease (IBD) and rheumatoid arthritis (RA) (16, 17). Lymphocyte subset count is a diagnostic biomarker of serious carbapenem-resistant Enterobacteriaceae infection (18). The lymphopenia observed in patients with acute pancreatitis can lead to bacterial invasion and thus secondary infection (12). In conclusion, cytokines and lymphocytes are involved in the pathophysiology of inflammation and inflammatory diseases. This means that cytokines and lymphocytes may have great potential for predicting post-PCNL SIRS. Notably, however, no studies have investigated the relationships between cytokines, lymphocyte subsets, and post-PCNL SIRS. Thus, the use of cytokines and lymphocyte subsets as biomarkers of post-PCNL SIRS was analyzed retrospectively in the current study.

## Materials and methods

### Study population

All adult patients who underwent PCNL at Tongji Hospital in China between October 2018 and January 2022 were retrospectively reviewed. The inclusion criteria were (1) age≥18 years (2), underwent PCNL, and (3) availability of preoperative computed tomography and blood test data. The exclusion criteria were (1) presence of infectious diseases or fever on admission (2); patients who were previously or currently diagnosed with malignant tumors (3); hematopathy, immune abnormalities, or was undergoing or had undergone immune-related therapy; and (4) use of antibiotics within 1 month before blood tests.

### Data collection

Pathological and clinical information were collected. All patients underwent routine blood routine, urine routine tests, and biochemistry analysis, and tests for serum cytokines and lymphocyte subsets at Tongji Hospital before surgery. Lymphocyte subsets were assessed by *via* flow cytometry, IL-6 assessed *via* Roche Electrochemiluminescence, and the rest of cytokines (IL-1β, IL-2 receptor [IL-2R], IL-8, IL-10, and TNF-α) by Simon Electrochemiluminescence. All patients also underwent computed tomography (CT) to confirm stone parameters. The stone burden was estimated using Ackermann’s formula (19). Staghorn stones are large branching stones that fill part of all the renal pelvis and renal calyces, and they can be complete or partial depending on the level of occupancy of the collecting system.

### Surgical technique and treatment strategy

All patients underwent corresponding preoperative, intraoperative, and postoperative treatment according to the latest European Association of Urology guidelines (20). Ceftriaxone 1 g i.v. was administered during anesthetic induction in patients with a negative urine culture. In patients with positive preoperative urine culture, antibiotics were given 5 days before surgery and during induction of anesthesia according to the results of the antibiotic map (21). All operations were performed by two experienced surgeons skilled in PCNL in our hospital. The general surgical process is as follows. A ureteral catheter was placed in patients under lithotomy position after general anesthesia. Under the prone position, the optimal percutaneous access was established by piercing the appropriate renal calyx, usually the middle and posterior kidney calyces, through the guidance of ultrasound. In addition, before a 20-Fr sheath could be placed, the fascial dilator was used to dilate the percutaneous track. Afterwards, the perfusion flow was set to about 400 ml/min and a holmium laser was used for disintegrating stones. It is worth noting that the nephroscope used in the surgery was 14 Fr. Last, an 18-Fr nephrostomy tube and a 6-Fr double-J stent were routinely placed according to standard procedure. The operative time was defined as the time from the placement of the ureteral catheter to the end of the placement of the nephrostomy tube. Nephrostomy stent was clamped on the second postoperative day and removed after 24 h if there was mild or no pain. The double-J stent was removed by flexible cystoscopy with local anesthesia 14 days after surgery. The follow-up evaluation was performed 2–3 months after PCNL, including renal function, routine urine test, and stone-free status. Patients who need a second stage of surgery would have a second-stage surgery 2 weeks or more after discharge.

### Endpoints

The primary endpoint of the study was the development of post-PCNL SIRS during postoperative hospitalization. All patients were routinely monitored for 2 days after surgery, and monitoring time was extended if necessary. Patients who met two or more of the following criteria were deemed to have developed SIRS [17]: (1) body temperature >38°C or <36°C; (2) heart rate >90 bpm; (3) respiratory rate >20 breaths/min or PaCO_2_ <32 mmHg; (4) white blood cell count >12 × 10^9^ cells/L or < 4 × 10^9^ cells/L. The secondary endpoints were the occurrence of sepsis, severe sepsis, and septic shock (9).

### Statistical analysis

Statistical analysis was performed using SPSS Software (version 26) and the R programming language (version 4.1.1). Data were reported as medians, quartiles, or percentages. Categorical variables were analyzed by using the chi-square test. Independent t-sample tests were performed on continuous variables that conformed to the normal distribution, and non-parametric tests were performed on variables that did not conform to the normal distribution. To identify independent risk factors for post-PCNL SIRS, variables with *p*-values <0.05 in univariate analysis were included in multivariate analysis conducted *via* forward stepwise selection. Adjusted 5% confidence intervals (CIs) and odds ratios (ORs) were calculated. All *p*-values were two-sided, and a *p*-value <0.05 was considered statistically significant.

## Results

The study included 154 patients who underwent one-stage PCNL for upper urinary tract stones. Among them, 50 (32.5%) patients developed post-PCNL SIRS and 4 (2.6%) patients developed post-PCNL sepsis. Compared with the non-SIRS group, the characteristics of the SIRS group included positive urine culture, high IL-2R, low CD3**^+^** cells, low CD3**^+^** CD4**^+^** cells, long operation times, low estimated glomerular filtration rate, and high WBC counts (**Tables 1**, **2**).

**Table 1:** Basic characteristics data in the non-SIRS group and the SIRS group.

Variables	Non-SIRS (*n* = 104)	SIRS (*n* = 50)	*p* value
**Demographics**			
Age, median (IQR), (years)	50.50 (39.50–57.75)	53.00 (44.00–57.25)	0.595
**Male**, Gender, *n* (%)	65.00 (62.50%)	30.00 (60.00%)	0.765
BMI, median (IQR)	23.58 (20.74–25.92)	23.39 (20.37–25.00)	0.993
**Clinical characteristics**			
Diabetes, *n* (%)	8.00 (7.69%)	8.00 (16.00%)	0.114
Hypertension, *n* (%)	33.00 (31.73%)	16.00 (32.00%)	0.973
Operation time, median (IQR), (min)	85.50 (62.00–103.75)	102.50 (85.00–130.25)	< 0.001
Stone burden, median (IQR),(mm^2^)	162.00 (80.00–289.00)	218.50 (96.75–399.00)	0.158
Staghorn calculi, *n* (%)	17.00 (16.35%)	11.00 (22.00%)	0.394
Stone side, *n* (%)			0.575
Left	38.00 (36.64%)	14.00 (28.00%)	
Right	39.00 (37.50%)	21.00 (42.00%)	
Bilateral	27.00 (25.96%)	15.00 (30.00%)	
Hydronephrosis, *n* (%)			0.575
Yes	79.00 (75.96%)	40.00 (80.00%)	
No	25.00 (24.04%)	10.00 (20.00%)	
**Routine blood tests**			
WBC, median (IQR), (10^9^/L)	5.78 (4.82–6.74)	6.15 (5.11–7.55)	0.005
Lymphocytes, median (IQR), (10^9^/L)	1.82 (1.39–2.18)	1.81 (1.47–2.32)	0.567
Neutrophils, median (IQR), (10^9^/L)	3.32 (2.58–4.21)	3.42 (2.23–4.45)	0.627
Hemoglobin, median (IQR), (g/L)	132.00 (121.00–148.00)	133.50 (117.00–140.00)	0.384
Total protein, median (IQR), (g/L)	67.80 (63.63–71.85)	68.70 (62.48–72.75)	0.919
Albumin, median (IQR), (g/L)	39.85 (37.28–42.28)	39.40 (36.45–41.50)	0.228
Globulin, median (IQR), (g/L)	27.05 (25.18–30.20)	28.95 (23.88–31.95)	0.422
Albumin-globulin ratio	1.46 (1.28–1.62)	1.39 (1.28–1.61)	0.230
**Kidney function**			
Creatinine, median (IQR), (µmol/L)	82.00 (69.00–99.75)	93.00 (67.25–133.00)	0.116
BUN, median (IQR), (mol/L)	5.77 (4.79–6.38)	5.55 (4.55–7.55)	0.255
Uric acid, median (IQR), (µmol/L)	337.50 (282.25–418.00)	371.00 (264.25–467.25)	0.201
eGFR, median (IQR), (mL/min)	87.15 (67.23–103.18)	82.80 (49.95–99.98)	0.052
**Urine examination**			
**Hematuria, *n* (%)**			0.505
positive	60.00 (57.69%)	26.00 (55.00%)	
negative	44.00 (42.31%)	24.00 (45.00%)	
**Urine leukocytes, *n* (%)**			0.406
positive	55.00 (52.88%)	30.00 (60.00%)	
negative	49.00 (47.12%)	20.00 (40.00%)	
**Urine protein, *n* (%)**			0.073
positive	25.00 (24.04%)	19.00 (38.00%)	
negative	79.00 (75.96%)	31.00 (62.00%)	
**Urine nitrite, *n* (%)**			0.003
positive	6.00 (5.77%)	11.00 (22.00%)	
negative	98.00 (94.23%)	39.00 (78.00%)	
**Urine nitrite, *n* (%)**			0.215
positive	3.00 (2.88%)	4.00 (8.00%)	
negative	101.00 (97.12%)	46.00 (92.00%)	

WBC, white blood cells; BUN, blood urea nitrogen; eGFR, estimated glomerular filtration rate.

**Table 2:** Cytokine and lymphocyte subsets in the SIRS group and the non-SIRS group.

Variables	Non-SIRS (*n* = 104)	SIRS (*n* = 50)	*p* value
IL-1β > 5 pg/mL, *n* (%)	40(38.46%)	19(38.00%)	0.956
IL-2R, median (IQR), U/mL	388.00(302.25-531.00)	507.50(388.00-733.75)	< 0.001
IL-6 ,median (IQR), pg/mL	4.21(1.00-10.58)	6.67 (1.00-13.99)	0.275
IL-8, median (IQR), pg/mL	13.65(9.70-28.25)	18.75(11.08-32.58)	0.376
IL-10 > 5 pg/mL, *n* (%)	6.00(5.77%)	1.00(2.00%)	0.429
TNF-α, median (IQR), pg/mL	9.90(7.90-15.85)	13.05(8.25-18.10)	0.347
Percentage of CD3**^+^** cells, median (IQR), %	74.86%(68.81%-78.96%)	69.48%(65.27%-75.30%)	0.003
CD3**^+^** cells, median (IQR), 1/µL	1255.00(956.00-1624.50)	1167.00(1002.25-1607.50)	0.517
Percentage of CD19**^+^** cells, %	12.01%(8.89%-16.67%)	13.39%(9.61%-16.98%)	0.25
CD19**^+^** cells, median (IQR), 1/µL	202.00(136.25-281.75)	224.00(132.75-338.50)	0.237
Percentage of CD3**^+^** CD4**^+^** cells, %	45.44%(39.94%-53.36%)	42.89%(37.35%-49.81%)	0.05
CD3**^+^** CD4**^+^** cells, median (IQR), 1/µL	782.00(597.00-999.50)	755.00(561.50-931.00)	0.335
Percentage of CD3**^+^** CD8**^+^** cells, median (IQR), %	21.89%(18.17%-27.27%)	22.09%(18.85%-26.92%)	0.894
CD3**^+^** CD8**^+^** cells, median (IQR), 1/µL	392.00 (283.25-531.75)	365.50(283.25-545.00)	0.879
Percentage of CD3- CD56**^+^** cells, %	12.06%(8.22%-16.97%)	13.81%(9.69%-19.66%)	0.307
CD3^-^CD56**^+^** cells, median (IQR), 1/µL	211.00(128.00-316.75)	230.00(150.00-332.75)	0.774
Percentage of TBNK cells, %	99.43%(99.07%-99.64%)	98.94%(99.45%-99.71%)	0.949
TBNK cells, median (IQR), 1/µL	1749.50(1392.50-2120.00)	1661.00(1407.75-2229.25)	0.86
CD4^+^/CD8^+^, median (IQR), %	2.12(1.51-2.72)	2.05(1.45-2.70)	0.421
CD3**^+^** CD4**^+^** CD28**^+^** cells, median (IQR), %	96.28%(92.93%-98.50%)	97.10%(91.94%-99.47%)	0.336
CD3**^+^** CD8**^+^** CD28**^+^** cells, median (IQR), %	67.54%(53.22%-76.80%)	66.36%(55.25%-80.41%)	0.416
CD3**^+^** HLA-DR**^+^** cells, median (IQR), %	16.80%(13.07%-21.86%)	16.82%(11.77%-21.39%)	0.578
CD3**^+^** CD8**^+^** HLA-DR**^+^** cells, median (IQR), %	40.65%(28.15%-50.92%)	40.08%(28.67%-48.63%)	0.519
CD3**^+^** CD4**^+^** CD45RO**^+^** cells, median (IQR), %	68.30%(56.20%-75.26%)	68.60%(57.98%-78.55%)	0.496
CD3**^+^** CD4**^+^** CD25**^+^** CD127^low^ cells, median (IQR), %	4.22%(3.31%-4.89%)	4.10%(3.47%-4.63%)	0.751
CD3**^+^** CD4**^+^** CD25**^+^** CD127^low^CD45RA**^+^** cells, median (IQR), %	0.98%(0.65%-1.36%)	0.85%(0.56%-1.30%)	0.479
CD3**^+^** CD4**^+^** CD25**^+^** CD127^low^CD45RO**^+^** cells, median (IQR), %	3.05%(2.48%-3.59%)	3.01%(2.44%-3.67%)	0.985

IL-1β, interleukin 1β; IL-2R, interleukin 2 receptor; IL-6, interleukin 6; IL-8, interleukin 8; IL-10, interleukin 10; TNF-α, tumor necrosis factor α; TBNK cells, CD3^+^ cells, CD19^+^ cells, and CD3^-^CD56^+^ cells.

In univariate analysis, five factors were identified as potential risk factors for post-PCNL SIRS; operation time (*p* = 0.001), urine culture (*p* = 0.005), IL-2R (*p* = 0.001), percentage of CD3^+^ cells (*p* = 0.004), WBC count (*p* = 0.009) (**Table 3**). These variables were included in multivariate regression analysis. In that regression analysis, positive urine culture (*p* = 0.048; OR, 3.556; 95% CI, 1.009–12.604), long operation time (*p* = 0.027; OR, 1.011; 95%CI, 1.001–1.022), high IL-2R (*p* = 0.018; OR, 1.002; 95%CI, 1.000–1.004), low percentage of CD3^+^ cells (*p* = 0.006; OR, 0.931; 95%CI, 0.885–0.980), high WBC count (*p* = 0.044; OR, 1.282; 95%CI, 1.007–1.632) were independent risk factors for post-PCNL SIRS.

**Table 3:** Univariate and multivariate analyses of risk factors for SIRS.

Variables	Univariate analysis		Multivariate analysis	
	OR (95% CI)	*p* value	OR (95% CI)	*p* value
Operation time	1.016 (1.007–1.026)	0.001	1.011 (1.001–1.022)	0.027
Urine culture	4.607 (1.593–13.319)	0.005	3.566 (1.009–12.604)	0.048
IL-2R	1.003 (1.001–1.004)	0.001	1.002 (1.000–1.004)	0.018
Percentage of CD3**^+^** cells	0.937 (0.896–0.979)	0.004	0.931 (0.885–0.980)	0.006
WBC Count	1.345 (1.078–1.678)	0.009	1.282 (1.007–1.632)	0.044
Percentage of CD3**^+^** CD4**^+^** cells	0.965 (0.926–1.005)	0.085		

WBC, white blood cells; IL-2R, interleukin 2 receptor.

**Figure 1:**
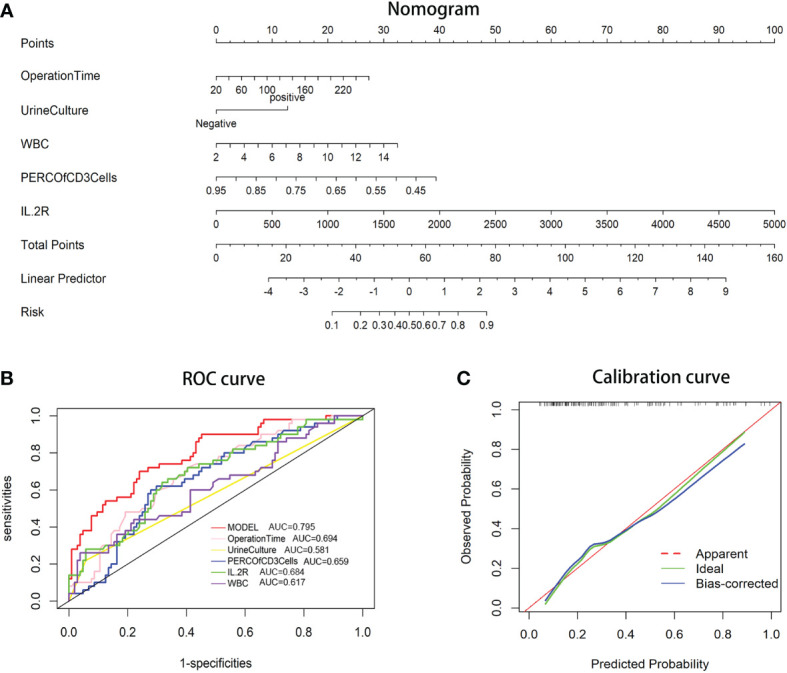
**(A)** Nomogram for patients predicting post-PCNL SIRS. Urine culture, operation time, percentage of CD3**^+^** cells, IL-2R, and WBC count are marked as “points.” Total points by adding the four points can predict SIRS rate. **(B)** Receiver operating characteristic (ROC) curve. The area under curve (AUC) for the model is 0.795, which showed a favorable ability of discrimination. **(C)** Calibration curve. Calibration curve shows good fitting of model.

A predictive nomogram based on the above-described five risk factors was generated to calculate the probability of occurrence of post-PCNL SIRS (**Figure 1A**). The total score was acquired by adding the scores of the independent risk factors. The incidence rate of post-PCNL SIRS was calculated from the incidence corresponding to the total score. The calibration curve exhibited a favorable fitting of the model (**Figure 1B**). Based on multivariate regression analysis, using post-PCNL SIRS as the endpoint, receiver operating characteristic (ROC) curves of the five independent risk factors for post-PCNL SIRS were performed (**Figure 1C**). In ROC curve analysis, the model based on all five factors had a higher under-the-curve (AUC) (AUC=0.795; 95%CI, 0.721–0.869) compared with operation time (AUC=0.694; 95%CI, 0.608–0.780), IL-2R (AUC=0.684; 95%CI, 0.594–0.774), urine culture (AUC=0.581; 95%CI, 0.519–0.643), percentage of CD3^+^ cells (AUC=0.659; 95%CI, 0.570–0.748), WBC count (AUC = 0.617; 95%CI, 0.520–0.714).

## Discussion

The rate of post-PCNL SIRS ranges from 11% to 35% in previous studies (22), and in the current study, the SIRS rate reached 32.5% (50/154). Five independent preoperative risk factors were identified *via* multivariate analysis: long operation time, positive urine culture, high IL-2R, low CD3^+^ cells, and high WBC count. A nomogram was established using those risk factors. The resulting ROC curves yielded an AUC curve of 0.795, indicating pleasurable discrimination. The calibration curve indicated a favorable fitting of the model.

In the present study, operation time was determined as an independent risk factor for SIRS. PCNL may cause excessive renal pelvic pressure, which may cause pyelotubular, pyelolymphatic, and pyelovenous backflow, which may in turn lead to the entry of bacteria into circulation (9). Zhong et al. (23) found that when renal pelvic pressure reached or exceeded 20 mmHg, the occurrence rate of postoperative fever increased. They also reported that as the time taken for renal pelvic pressure to reach or exceed 30 mmHg was prolonged, the postoperative fever rate increased. Longer operation times may result in increased time for renal pelvic pressure to exceed a certain threshold, in turn increasing the likelihood of bacteria and endotoxin entering the circulation. A low estimated glomerular filtration rate (eGFR) was identified as a characteristic of the SIRS group in the present study. Preoperative creatinine levels have been shown to correlate with postoperative sepsis (9). Creatinine and eGFR are important indicators of kidney function, and they may both be associated with postoperative SIRS. Studies on renal function and post-PCNL SIRS are needed to confirm this.

In previous studies, positive urine culture has been associated with post-PCNL SIRS (24, 25), and this was also the case in the present study. Positive urine culture indicates the presence of a preoperative urinary tract infection. During PCNL, high intrarenal pressure may introduce toxins and bacteria from urine into the circulation (26). Similar to urine culture, stone culture has also been identified as an independent risk factor for post-PCNL SIRS. However, the results of the stone culture will not be known until 2 days or more after the test, and SIRS often occurs several hours to a few days after surgery. This limits the clinical usefulness of stone culture. Thus, it was not included in the present study. For the same reason, stone components analysis was not included in the present study.

IL-1β, IL-6, IL-8, and TNF-α are involved in the pathogenesis of many inflammatory diseases including rheumatoid arthritis, psoriatic arthritis, and IBD (17). Qi et al. (27) found that IL-6 was a useful biomarker of urosepsis. IL-2R has been shown to be closely related to immune function, and studies have demonstrated the associations between IL-2R and infections, such as pleural tuberculosis and the human T-cell leukemia virus (28, 29). To our knowledge, the present study is the first to investigate the associations between post-PCNL SIRS and IL-2R. In the current study, high IL-2R was an independent risk factor for post-PCNL SIRS. IL-2 regulates T- cell differentiation, function, effector, and memory responses by interacting with three classes of IL-2R (IL-2Rα, IL-2β, and IL-2γ), each with different affinities for IL-2 (30). If IL-2R disappears for any reason, the ability of T cells to respond to IL-2 is compromised. Increased IL-2R may indicate that the immune system is in an abnormal state, which may explain the results in the present study.

There are numerous reports that show that reduced total T cells (CD3**^+^** cells) and reduced WBC counts are associated with infectious diseases and sepsis (11, 31, 32). In the serum of patients with coronavirus disease 2019, particularly critically ill patients, CD3**^+^** cell counts are significantly reduced (33). In the current study, a low percentage of CD3^+^ cells was an independent risk factor for post-PCNL SIRS. A decrease in the proportion of T cells may indicate compromised cellular immune function, which is insufficient to resist the invasion of harmful factors during PCNL. An elevated WBC count may represent a prodromal period of infection. Thus, patients with a low percentage of CD3^+^ cells are prone to SIRS after surgery. CD3**^+^** CD4**^+^** cells are also known as T- helper cells, and the subsets of T- helper cells are involved in the pathogenesis of infectious and inflammatory diseases (34). However, subsets of T- helper cell counts that might be associated with SIRS were not performed in this study. Further studies are required to identify the role of CD3**^+^** CD4**^+^** cells in SIRS.

The current study indicates for the first time that IL-2R and the percentage of CD3^+^ cells can be used as diagnostic markers of post-PCNL SIRS. A preoperative nomogram based on five independent risk factors for post-PCNL SIRS was constructed. This may help doctors identify high-risk patients in a timely manner before surgery so that they can take a more prudent approach to these patients, such as performing surgery on high-risk patients by doctors proficient in PCNL, controlling operation time, and rigorous perioperative monitoring. There are a number of studies that have identified risk factors for SIRS after PCNL such as urine culture, stone culture, and surgical time, which are important for clinical practice. This study analyzed the relationship between the function of the immune system before surgery, which is related to cytokines, lymphocyte subsets, and SIRS after PCNL. Our study may provide new horizons for a future related study.

The current study had some limitations. Five independent risk factors for post-PCNL SIRS were identified, but the reliability of these predictors should be tested by other researchers. The specific mechanisms resulting in associations between post-PCNL and IL-2R and CD3^+^ cells remain unclear. Biomedical experiments at the cellular level are required to verify the specific molecular mechanisms involved. Procalcitonin and C-reactive protein are considered predictive biomarkers of post-PCNL SIRS (35) but are usually assessed after surgery at our institution. Hence, C-reactive protein and procalcitonin were not included in this study. Moreover, the present study may have potential selection bias due to its retrospective nature and the fact that it was conducted at a single center. Future comprehensive multicenter studies with larger sample sizes should be performed to elucidate the clinical significance of the model and the preoperative management of high-risk patients.

## Conclusion

The present study indicated for the first time that IL-2R and the percentage of CD3**^+^** cells are independent risk factors for post-PCNL. A new nomogram was established to predict post-PCNL SIRS. The results of the study suggest that the nomogram may help clinicians to identify high-risk patients and conduct corresponding therapy in advance.

## Data availability statement

The original contributions presented in the study are included in the article/**Supplementary Material**. Further inquiries can be directed to the corresponding authors.

## Ethics statement

The studies involving human participants were reviewed and approved by Tongji Hospital. The ethics committee waived the requirement of written informed consent for participation.

## Author contributions

ZC and DX, project development, and manuscript editing. KT, project development, and manuscript writing. YH, data analysis, and manuscript writing. YT, EP, and QH, data collection. HS, data analysis. XL, manuscript writing. All authors contributed to the article and approved the submitted version.

## Funding

Our research was funded by the National Natural Science Foundation of China (Nos. 81900645, 82170779, and 82270804), the Natural Science Foundation of Hubei Province (2021CFB366), 2019 Wuhan Yellow Crane Talent Program (Outstanding Young Talents), and the Tongji Hospital (HUST) Foundation for Excellent Young Scientist (No. 2020YQ15).

## Conflict of interest

The authors declare that the research was conducted in the absence of any commercial or financial relationships that could be construed as a potential conflict of interest.

## Publisher’s note

All claims expressed in this article are solely those of the authors and do not necessarily represent those of their affiliated organizations, or those of the publisher, the editors and the reviewers. Any product that may be evaluated in this article, or claim that may be made by its manufacturer, is not guaranteed or endorsed by the publisher.
